# Transcriptional regulation of the CRK/DUF26 group of Receptor-like protein kinases by ozone and plant hormones in Arabidopsis

**DOI:** 10.1186/1471-2229-10-95

**Published:** 2010-05-25

**Authors:** Michael Wrzaczek, Mikael Brosché, Jarkko Salojärvi, Saijaliisa Kangasjärvi, Niina Idänheimo, Sophia Mersmann, Silke Robatzek, Stanisław Karpiński, Barbara Karpińska, Jaakko Kangasjärvi

**Affiliations:** 1 Plant Biology Division, Department of Biosciences, University of Helsinki, FI-00014 Helsinki, Finland; 2 Department of Biology, University of Turku, FI-20014 Turku, Finland; 3 Max-Planck Institute for Plant Breeding Research, Carl-von-Linné-Weg 10, DE-50829 Cologne, Germany; 4 The Sainsbury Laboratories, Norwich Research Park, Colney Lane, NR4 7UH, Norwich, UK; 5 Department of Plant Genetics, Breeding and Plant Biotechnology, Warsaw University of Life Sciences, Nowoursynowska st. 159, PL 02-776, Warsaw, Poland; 6 Department of Life Sciences, Södertörn University College, SE-141 89 Huddinge, Sweden

## Abstract

**Background:**

Plant Receptor-like/Pelle kinases (RLK) are a group of conserved signalling components that regulate developmental programs and responses to biotic and abiotic stresses. One of the largest RLK groups is formed by the Domain of Unknown Function 26 (DUF26) RLKs, also called Cysteine-rich Receptor-like Kinases (CRKs), which have been suggested to play important roles in the regulation of pathogen defence and programmed cell death. Despite the vast number of RLKs present in plants, however, only a few of them have been functionally characterized.

**Results:**

We examined the transcriptional regulation of all *Arabidopsis CRKs *by ozone (O_3_), high light and pathogen/elicitor treatment - conditions known to induce the production of reactive oxygen species (ROS) in various subcellular compartments. Several *CRKs *were transcriptionally induced by exposure to O_3 _but not by light stress. O_3 _induces an extracellular oxidative burst, whilst light stress leads to ROS production in chloroplasts. Analysis of publicly available microarray data revealed that the transcriptional responses of the *CRKs *to O_3 _were very similar to responses to microbes or pathogen-associated molecular patterns (PAMPs). Several mutants altered in hormone biosynthesis or signalling showed changes in basal and O_3_-induced transcriptional responses.

**Conclusions:**

Combining expression analysis from multiple treatments with mutants altered in hormone biosynthesis or signalling suggest a model in which O_3 _and salicylic acid (SA) activate separate signaling pathways that exhibit negative crosstalk. Although O_3 _is classified as an abiotic stress to plants, transcriptional profiling of CRKs showed strong similarities between the O_3 _and biotic stress responses.

## Background

Receptor-like/Pelle kinases (RLKs) are important components in the regulation of plant development, hormone signalling, abiotic, and biotic stress responses in plants. RLKs are serine-threonine protein kinases that typically contain a signal peptide, a variable extracellular domain, a transmembrane region, and a conserved intracellular protein kinase domain. The extracellular ligand-binding domain perceives signals and is commonly used to classify RLKs into distinct subgroups [1]. The RLKs are one of the largest gene families in *Arabidopsis *with more than 600 members, [1-4], but only relatively few of them, mostly leucine-rich repeat RLKs (LRR-RLK), have been functionally characterized. CLAVATA1, a LRR-RLK, binds the small extracellular protein CLAVATA3 to regulate meristem proliferation [5]. FERONIA (a member of a previously uncharacterized group of RLKs) is central to the regulation of male-female interactions during pollen tube reception in *Arabidopsis *[6] and in *Brassica *the S-locus Receptor Kinase and its ligand are critical determinants of self-incompatibility [7,8]. In *Arabidopsis*, ERECTA (a LRR-RLK) is a multifaceted regulator of development and physiological processes as well as environmental responses [9]. BRASSINOSTEROID INSENSITIVE 1 (BRI1, a LRR-RLK) binds the plant hormone brassinosteroid and dimerizes with BRI1-ASSOCIATED RECEPTOR KINASE 1/SOMATIC EMBRYOGENESIS RECEPTOR KINASE 3 (BAK1/SERK3) [10,11]. BAK1 also inducibly dimerizes with the RLK FLAGELLIN SENSITIVE 2 (FLS2, a LRR-RLK), which recognizes bacterial flagellin and is important in plant immunity [12,13]. Other RLKs contributing to pathogen recognition include EFR (the *Arabidopsis *receptor for EF-Tu) and rice Xa21 (a LRR-RLK), which recognizes a sulfonated peptide produced by the pathogen *Xanthomonas oryzae *pv. *oryzae *[14-18].

The DUF26 (Domain of Unknown Function 26; PFAM domain PF01657) RLKs, also known as Cysteine-rich RLKs (CRKs), form a large subgroup of the RLK family with more than 40 members [1,19]. The extracellular region of the protein contains two copies of the DUF26 domain which has four conserved cysteines (three of them form the motif C-8X-C-2X-C) that may form disulphide bridges as potential targets for thiol redox regulation. The *CRKs *are transcriptionally induced by oxidative stress, pathogen attack and application of salicylic acid (SA) [19-22]. Accordingly several members of the CRK subgroup of RLKs are involved in the regulation defence reactions and cell death in *Arabidopsis *leaves. Constitutive over-expression of CRK5 led to increased resistance to the virulent bacterial pathogen *Pseudomonas syringae *pv. tomato DC3000 but also to enhanced growth of the plant leaves [22]. Over-expression of CRK4, CRK5, CRK19 and CRK20 by a chemically inducible promoter, on the other hand, caused cell death [19,22]. Genetic analysis suggested that CRK5 regulated cell death independently of SA [22]. Conversely the enhanced resistance to *Pseudomonas *upon overexpression of CRK13 required increased SA levels [23].

Reactive oxygen species (ROS) have been established as important signalling molecules for inter- and intracellular communication in plants, animals and yeast [24-26]. ROS are produced in strictly defined locations in reponse to specific stimuli [25]. Pathogen infection rapidly induces an extracellular oxidative burst while light stress and specific chemicals, including paraquat and norflurazon, induce ROS production in the chloroplast [27-29]. Plant cells can differentiate between the type and localization of ROS resulting in very specific responses. Furthermore, ROS production in specific cellular compartments can have impact on ROS generation and signalling in other locations [30,31]. This crosstalk is likely accomplished through interplay between separate signalling pathways rather than direct interaction of the ROS molecules themselves [30,31]. However, the molecular components and mechanisms involved are still poorly defined [31,32]. In addition, it is unknown how ROS are sensed and how specificity in ROS signalling is achieved. The gaseous molecule ozone (O_3_) induces a burst of ROS in the apoplast similar to the oxidative burst in plant-pathogen interactions [24]. Other similarities between O_3 _and pathogen infection include the production of SA and ethylene (ET) [24]. O_3 _is a convenient system to experimentally address the effects of apoplastic ROS since the plant is not exposed to other effector proteins or toxins which might induce defence responses. O_3 _permits the study of the apoplastic oxidative burst undisturbed by manual manipulation of the plant material.

Plant hormones are a group of unrelated small compounds which are central to signalling during environmental adaptation and developmental regulation [33,34]. SA, jasmonic acid (JA) and ET are viewed as the main hormonal determinants of plant pathogen defence [35,36]. Abscisic acid (ABA) modulates plant defence and is a negative regulator of SA responses [37]. In addition, ABA is a key regulator of the high light response [38]. The interaction of hormone and ROS signalling is well documented. ROS can induce cell death in a SA-dependent and independent manner [24]. Cell death and ROS induce ET synthesis, which feeds into a positive forward amplification loop enhancing ROS production [39]. ROS-induced JA is critical in limiting cell death [24]. Thus, the successful outcome of a given response is not determined by one hormone, but is achieved through balance, interaction and constant recalibration of different plant hormones.

Despite extensive research on ROS signalling, the exact components mediating ROS signalling, ROS sensing, and perception in particular are still unknown. Here we have analysed transcriptional regulation and the involvement of hormonal signalling in regulating the expression of the whole *Arabidopsis CRK *gene subfamily by ROS. The effects of ROS production in different subcellular compartments was analysed by using O_3_- and light stress treated plant material and publicly available microarray data. We show that O_3_-induced transcriptional responses are blocked in the *defense, no death 1 *(*dnd1*) mutant, and they are altered in hormone biosynthesis or signalling mutants. Collectively this reveals alternate pathways in the regulation of ROS responses.

## Results

### CRK transcriptional response to O_3_

Several groups of RLKs are transcriptionally regulated in response to biotic stresses [40]. We identified several *CRKs *which were differentially regulated by O_3 _(MB and JK unpublished microarray data). These results suggest a strong transcriptional regulation of the *CRKs *during stress responses. Therefore we chose to investigate further the transcriptional regulation of the whole *CRK *subfamily by ROS.

According to Shiu and Bleecker [1], Chen *et al*. [19], and our analysis (see table 1 for nomenclature and reference), the *CRK *subfamily consists of 44 members. Previously two additional genes have been included, but *At4g11500 *(*DUF26 44*) was classified as a pseudogene in the current version of the *Arabidopsis *genome (TAIR9; http://www.arabidopsis.org[41]) and *At4g23170 *(*CRK9*) contains no identifiable extracellular domain, signal peptide or complete kinase domain; thus both genes were excluded from the analysis.

**Table 1:** Nomenclature of the CRKs/DUF26 RLKs.

CRK Nomenclature	AGI Code	DUF26 Nomenclature
CRK1	At1g19090	DUF26 40

CRK2	At1g70520	DUF26 41

CRK3	At1g70530	DUF26 39

CRK4	At3g45860	DUF26 14

CRK5	At4g23130	DUF26 13

CRK6	At4g23140	DUF26 6

CRK7	At4g23150	DUF26 8

CRK8	At4g23160	DUF26 7

CRK10	At4g23180	DUF26 9

CRK11	At4g23190	DUF26 4

CRK12	At4g23200	DUF26 1

CRK13	At4g23210	DUF26 25

CRK14	At4g23220	DUF26 2

CRK15	At4g23230	DUF26 36

CRK16	At4g23240	DUF26 22

CRK17	At4g23250	DUF26 21

CRK18	At4g23260	DUF26 20

CRK19	At4g23270	DUF26 15

CRK20	At4g23280	DUF26 11

CRK21	At4g23290	DUF26 23

CRK22	At4g23300	DUF26 5

CRK23	At4g23310	DUF26 12

CRK24	At4g23320	DUF26 24

CRK25	At4g05200	DUF26 10

CRK26	At4g38830	DUF26 30

CRK27	At4g21230	DUF26 43

CRK28	At4g21400	DUF26 28

CRK29	At4g21410	DUF26 29

CRK30	At4g11460	DUF26 19

CRK31	At4g11470	DUF26 17

CRK32	At4g11480	DUF26 18

CRK33	At4g11490	DUF26 16

CRK34	At4g11530	DUF26 3

CRK36	At4g04490	DUF26 31

CRK37	At4g04500	DUF26 32

CRK38	At4g04510	DUF26 35

CRK39	At4g04540	DUF26 34

CRK40	At4g04570	DUF26 33

CRK41	At4g00970	DUF26 26

CRK42	At5g40380	DUF26 38

CRK43	At1g70740	DUF26 37

CRK44	At4g00960	DUF26 27

CRK45	At4g11890	DUF26 45

CRK46	At4g28670	DUF26 42

Nomenclature of the CRK/DUF26 group of RLKs according to Chen et al. [19] and Shiu and Bleecker [1]. CRK35 was not listed in Chen et al. [19].

**Figure 1:**
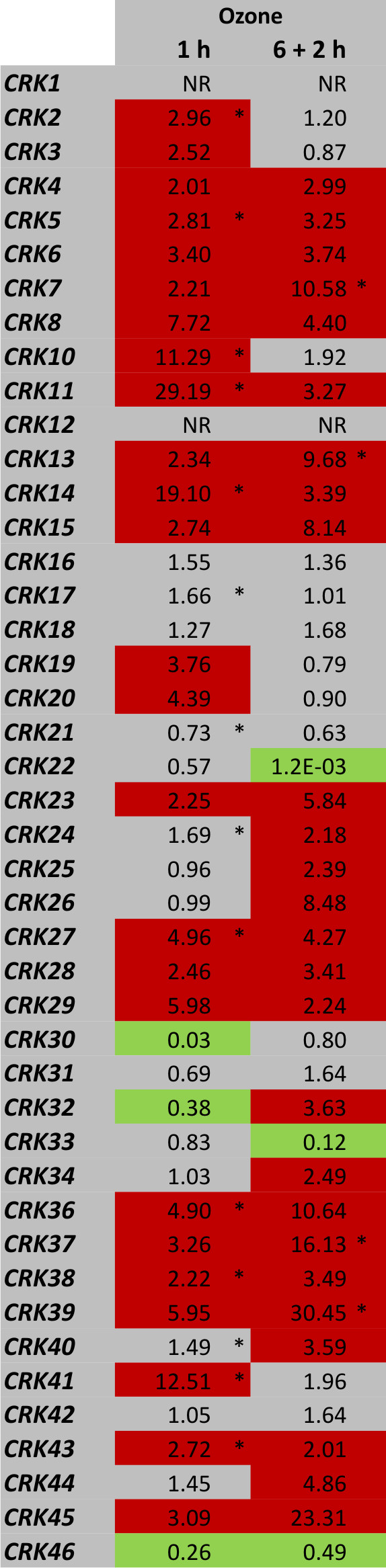
**Transcriptional regulation of the CRKs in response to O_3_**. Expression of all members of the *CRK *group of plant RLKs was analyzed by quantitative real-time RT-PCR (qPCR) in Col-0 plants exposed to 250 ppb O_3 _for 6 h. Samples were harvested at 1 or 8 h (6 h followed by 2 h recovery under clean air conditions) after the onset of the O_3 _treatment. Transcript levels were calculated by comparison of O_3_-exposed plants with corresponding control plants grown under clean air conditions harvested in parallel with the O_3_-treated plants. An expression level of one indicates no change in expression, increased expression is indicated by values larger than one while decreased expression is shown by values smaller than one. Increase in expression by 2-fold or higher is high-lighted in red and decrease in expression by 2-fold or more in green. NR - no reproducible data could be obtained for this gene. The experiment was repeated four times; fold change was calculated from the average normalized cycle difference of all biological repeats. Statistical significance (Benjamini-Hochberg FDR corrected p-value ≤ 0.1) is indicated with asterisks (see additional file 1).

**Figure 2:**
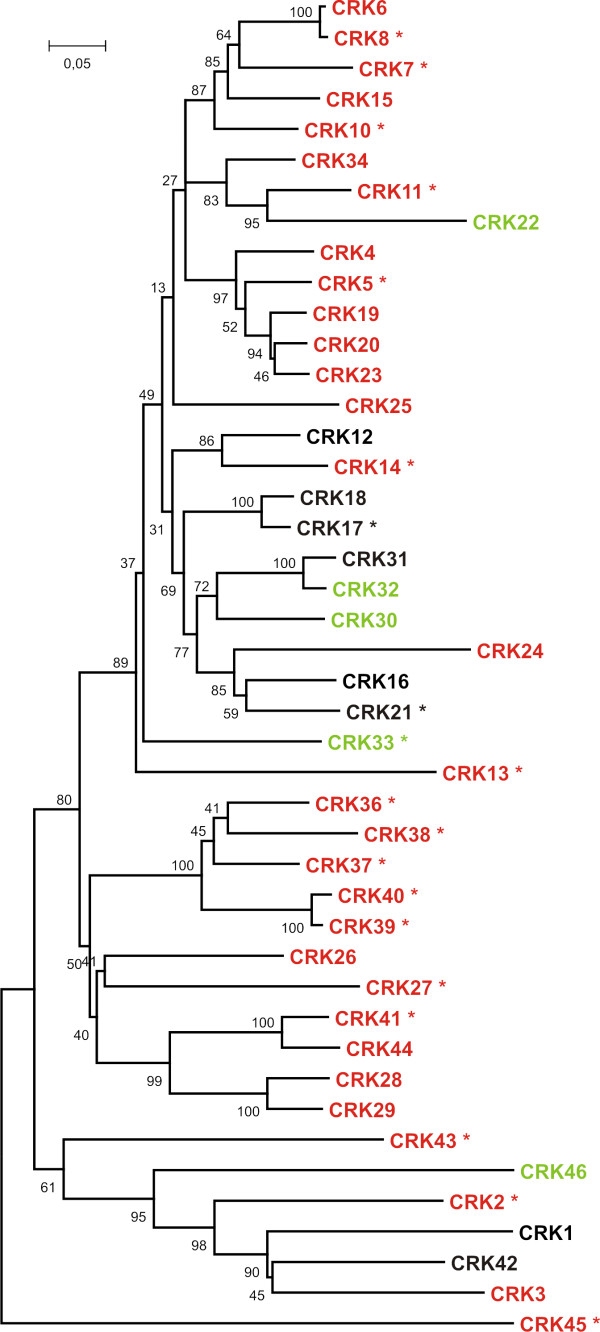
**Phylogenetic tree of the CRK kinase domains indicates that O_3 _regulation is distributed throughout group**. The kinase domains of all CRKs were aligned using ClustalW2 and a Neighbour-joining tree was constructed using MEGA4 [84]. DUF26 44 (At4g11500) and CRK9 (At4g23170) were not included in the analysis. Genes with increased expression by O_3 _treatment are indicated in red and genes with decreased expression in green (statistically significant changes are indicated by an asterisk).

We analysed the transcriptional responses of all the 44 *CRKs *to extracellular ROS produced by O_3 _by quantitative real-time RT-PCR (qPCR). Out of the 44 *CRKs*, 25 (nine with statistical significance FDR [False Discovery Rate]-corrected p-value ≤ 0.1; additional file 1) showed more than two-fold higher mRNA abundance after 1-hour exposure to O_3 _(Figure 1). After a 6-hour O_3 _exposure followed by a 2-hour recovery period, 26 *CRKs *exhibited a more than two-fold increase in expression (eight with statistical significance FDR-corrected p-value ≤ 0.1; additional file 1). Only *CRK22*, *CRK30*, *CRK32*, *CRK33 *and *CRK46 *showed decreased expression in response to O_3_-treatment. In order to analyze if transcriptional regulation after exposure to O_3 _was a feature of a single subset of the *CRKs*, the protein sequence of the kinase domain of all CRKs was aligned to construct a Neighbour-joining tree representing the relations between the members of the CRK group of RLKs (Figure 2). *CRKs *that were transcriptionally regulated in response to O_3 _are high-lighted. O_3_-regulated genes were distributed across the tree instead of forming a unique branch. However, closely related genes showed a tendency to share similar O_3 _expression patterns.

### CRK transcriptional response to light stress

**Figure 3:**
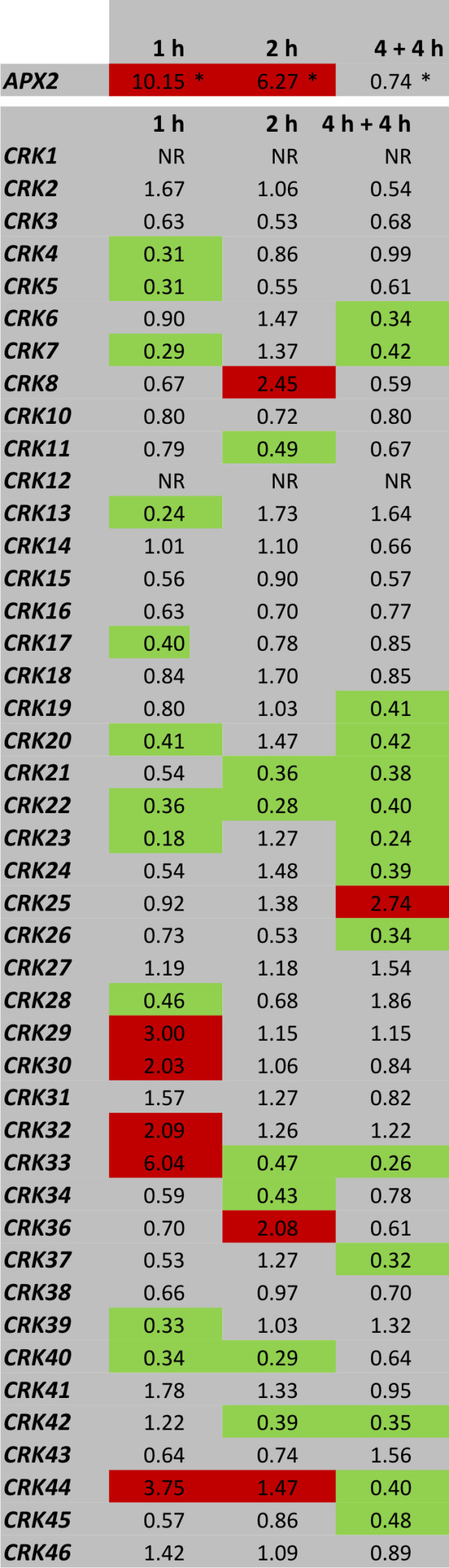
**Transcriptional downregulation of CRKs in response to light stress**. Expression of *APX2 *(a marker for light stress) and *CRKs *was analyzed by qPCR in plants after 1 h and 2 h exposure to light stress conditions and after 4 h light stress followed by 4 hours recovery at normal growth light conditions. Transcript levels were calculated by comparison of light stress-treated plants with corresponding control plants grown under normal light conditions. An expression level of one indicates no change of expression, increased expression is indicated by values larger than one while decreased expression is shown by values smaller than one. Increase of expression by 2-fold or higher is high-lighted in red and decrease in expression by 2-fold or more in green. NR - no reproducible data could be obtained for this gene. The experiment was repeated twice; fold change was calculated from the average normalized cycle difference of all biological repeats. Statistical significance (Benjamini-Hochberg FDR corrected p-value ≤ 0.1) is indicated with asterisks (see additional file 1).

To determine the effects of light stress-induced ROS production, we monitored the expression of *ASCORBATE PEROXIDASE 2 *(*APX2*), encoding a ROS scavenger and established marker for light-induced ROS production [42]. *APX2 *was strongly induced after 1- and 2-hour exposure to light stress conditions (Figure 3). In contrast to O_3 _(Figure 1), light stress led to rapid transcriptional repression of several CRKs (Figure 3). Twenty *CRKs *were transcriptionally repressed while only eight exhibited increased expression. However, the light-dependent regulation of the *CRKs *was not statistically significant. The lack of transcriptional induction in response to light stress corresponds to results from Lehti-Shiu *et al*. [40], who reported that the *CRKs *were transcriptionally strongly induced in response to biotic stimuli but the expression level decreased in response to abiotic stress (including heat, cold, drought and salt). Of the abiotic treatments, only UV-B, osmotic stress and wounding resulted in increased expression of CRKs [40].

### CRK transcriptional response to PAMPs is similar to the O_3 _response

**Figure 4:**
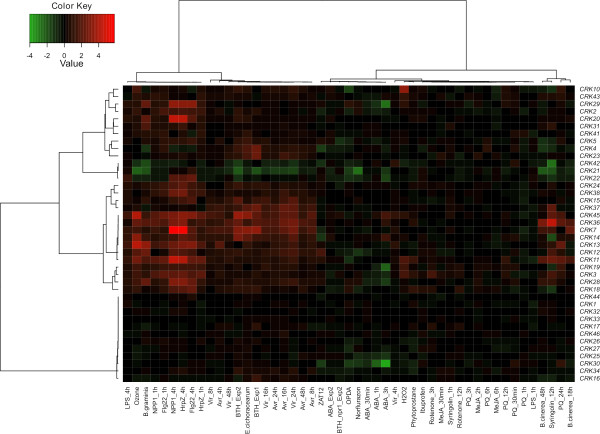
**Bayesian hierarchical clustering of the CRKs in abiotic and biotic stress experiments**. Biotic and abiotic stress data sets were down loaded from public databases and included O_3_, norflurazon, paraquat, BTH (benzothiadiazole S-methylester), various elicitors and pathogens (see materials and methods for complete details). Red and green indicate increased or decreased expression compared to untreated plants, respectively. The intensity of the colours is proportional to the absolute value of the fold difference.

To more broadly address transcriptional regulation of the *CRKs*, we analyzed and compared their expression profiles from publicly available Affymetrix chip data. Raw data files were obtained from several databases (see material and methods) and RMA (Robust Multi-Array Average) normalized. To take the sample variation into account, parametric bootstrapping combined with Bayesian hierarchical clustering [43] was applied. This results in a numerical measure of similarity between treatments and genes, which can be clustered hierarchically (Figure 4; for a related application, see [44]). The meta-analysis of the publicly available O_3 _microarray data revealed high overlap with our qPCR data; all eight genes with more than 3-fold increased expression in the publicly available array data exhibited increased expression in our qPCR analysis. Treatment with norflurazon (which increases singlet oxygen [^1^O_2_] in the chloroplast causing excess ROS production) led to decreased expression of four *CRKs*. Norflurazon blocks carotenoid biosynthesis and thus removes this quencher of the triplet chlorophyll and ^1^O_2_. Paraquat leads to superoxide  production in the chloroplast by transferring electrons from photosystem I to oxygen. The  is subsequently dismutated to H_2_O_2_. Paraquat had no effect on *CRK *expression with the exception of the latest time point tested (24 hr), whereupon five *CRKs *exhibited increased expression; four of which were also regulated in response to O_3_. However, at this time point paraquat had most likely induced cell death. H_2_O_2 _treatment selectively led to increased expression of a few *CRKs *which also displayed increased expression by O_3_. Rotenone (an inhibitor of mitochondrial electron transport causing elevated ROS production in mitochondria) had little impact on *CRK *expression; only *CRK3 *showed increased expression levels. Thus, the *CRK *expression profile triggered by O_3 _was not related to expression profiles established by other ROS treatments. Instead, the O_3_-triggered *CRK *expression profile clustered together with that provoked by several biotic and PAMP treatments, including *Blumeria graminis *var. *hordei *(*Bgh*), harpin Z (HrpZ), and the flagellin elicitor-active epitope flg22 (Figure 4).

Our qPCR analysis confirmed the changes caused by flg22 in the expression profile of the *CRKs *obtained from publicly available microarray data (Additional file 2 and Figure 4). Treatments with benzothiadiazole S-methylester (BTH; an active SA analog) resulted in two-fold or higher up-regulation of 12 *CRKs*, some of which also exhibited elevated expression in response to O_3_. Interestingly, in the *non-expressor of pathogenesis-related genes 1 *(*npr1*) mutant these genes were not regulated by BTH treatment (Figure 4), indicating that SA regulation of these genes was dependent on NPR1-mediated signalling. Application of methyl-jasmonate (MeJA) did not cause any major changes in *CRK *expression (Figure 4), whilst ABA treatment resulted in decreased expression of *CRK25*, *CRK30*, *CRK28*, *CRK29*, *CRK19*, *CRK21 *and *CRK22 *at late time points. Overall, the *CRK *expression profile in response to BTH clustered together with that triggered by O_3_, pathogen and PAMP treatments; whereas *CRK *transcriptional regulation upon ABA application clustered together with paraquat, norflurazon, rotenone and MeJA treatments (Figure 4).

Taken together, these results demonstrate that the *CRK *expression profile in response to O_3 _is not related to treatments which mediate ROS production in the chloroplast or the mitochondria. However, there is a substantial overlap between the transcriptional responses to O_3 _and pathogen infection/PAMP perception, which may be a result of apoplastic ROS commonly generated by all these stimuli.

### CRKs display different expression in hormone mutants

**Figure 5:**
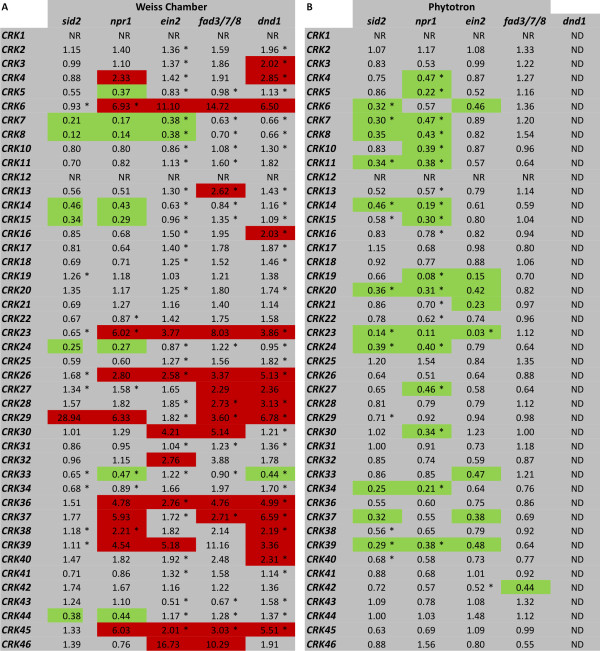
**Expression of CRKs is changed in hormone mutants**. The expression of all *CRKs *was analyzed by qPCR in the SA mutants *sid2 *and *npr1*, the ET mutant *ein2*, the JA mutant *fad3*/7/8 and the cell death mutant *dnd1 *by qPCR and compared to Col-0 under two different growth conditions. (A) Weiss chamber conditions. (B) Phytotron. Transcript levels were calculated by comparison between mutants and Col-0 grown under control conditions. An expression level of one indicates no change of expression, increased expression is indicated by values larger than one while decreased expression is shown by values smaller than one. Increase of expression by 2-fold or higher is high-lighted in red and decrease in expression by 2-fold or more in green. NR - no reproducible data could be obtained for this gene. ND - The *dnd1 *mutant did not grow in the phytotron. Fold-change is shown for the geometric mean of all biological repeats (n = 4). Statistically significance (Benjamini-Hochberg FDR corrected p-value ≤ 0.1) is indicated with asterisks (see additional file 1).

Altered transcriptional regulation of several *CRKs *has previously been shown following external application of the plant hormone SA or its active analog BTH (Figure 4 and [19]). In order to address the impact of hormone signalling on transcriptional regulation of *CRKs*, we used several mutants impaired in hormone biosynthesis and/or signalling. The *salicylic acid induction deficient 2 *(*sid2*) mutant is deficient in SA biosynthesis (due to a mutation in the SA biosynthesis gene *ISOCHORISMATE SYNTHASE 1 *[*ICS1*]), whilst *npr1 *is impaired in SA signalling. The *dnd1 *mutant fails to produce a hypersensitive response (HR), but has functional effector-triggered immunity, constitutive systemic resistance and accumulates elevated SA levels [45-47]. The *ethylene insensitive 2 *(*ein2*) mutant is deficient in ET signalling, and the *fatty acid desaturase 3/7/8 *(*fad3/7/8*) mutant is deficient in JA biosynthesis. We compared the transcript abundance of *CRKs *in these mutants to Col-0 wild type plants using qPCR. The obtained *Actin-2*-normalized threshhold cycle values (Ct) were compared between Col-0 wild type and the mutants. Several *CRKs *showed lower expression in *sid2 *and *npr1 *(Figure 5A). *CRK29 *displayed higher expression in *sid2 *and ten *CRKs *(three with statistical significance FDR-corrected p-value ≤ 0.1) exhibited higher expression in *npr1*. In the *ein2 *and *fad3/7/8 *mutants, for nine and twelve *CRKs*, respectively, expression levels were elevated as compared to wild type plants. Only *CRK7 *and *CRK8 *showed lower expression in *ein2*. Along with several other defects, *dnd1 *exhibits constitutive SA responses [48], which might be the cause for the increased transcript levels of 15 *CRKs *in *dnd1 *signalling -however, other regulatory mechanisms cannot be ruled out due to the pleiotropic nature of the mutant [48]. Expression of some *CRKs *was unaltered or displayed only subtle changes in the *sid2 *mutant, but was elevated in *npr1*, *ein2*, *fad3/7/8 *and *dnd1 *mutants (*CRK6*, *CRK23*, *CRK26*, *CRK36*, and *CRK45*). Interaction between hormone signalling pathways is an established phenomenon [24,37], and the *CRKs *above exemplify that altering the balance of SA, JA or ET response leads to altered gene expression.

To test the robustness of gene expression in this set of hormone mutants, we compared two different growth conditions. These differed in photoperiod, light composition and intensity, soil composition and humidity (see Materials and Methods for a detailed description of the differences in the growth conditions), subsequently referred to as Weiss chamber (Figure 5A) and Phytotron (Figure 5B). Notably, the *dnd1 *mutant did not grow under Phytotron conditions. The higher transcript abundance of *CRKs *in *ein2 *and *fad3/7/8 *observed in plants grown under Weiss chamber growth conditions was largely absent in plants grown under Phytotron growth conditions (Figure 5B). Moreover, the *CRKs *which showed higher gene expression in *npr1 *under Weiss chamber growth conditions, were unaltered (or had even reduced transcript levels) in the Phytotron. Taken together, these results indicate that hormones play a major role in the transcriptional regulation of many *CRKs*. However, environmental growth conditions also have a large impact on the extent of this regulation especially in soil grown plants [49,50].

#### O_3_-response of the CRKs in hormone mutants

**Figure 6:**
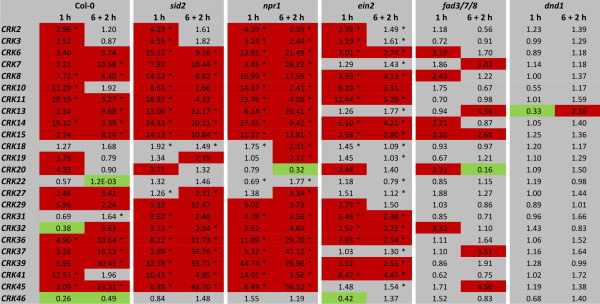
**O_3_-regulation of CRKs is different in hormone mutants**. The expression of 24 O_3_-regulated *CRKs *was analyzed by qPCR in Col-0 and *sid2*, *npr1*, *dnd1*, *ein2 *and *fad3*/7/8 exposed to 250 ppb O_3 _for 6 h. Samples were harvested at 1 or 8 h (6 h plus 2 h recovery under clean air conditions) after the onset of the O_3 _treatment. Transcript levels for Col-0 or each mutant line were calculated by comparison of O_3_-exposed plants with corresponding control plants of the same line grown under clean air conditions. An expression level of one indicates no change of expression, increased expression is indicated by values larger than one while decreased expression is shown by values smaller than one. Increase of expression by 2-fold or higher is high-lighted in red and decrease in expression by 2-fold or more in green. NR - no reproducible data could be obtained for this gene. The experiment was repeated four times; fold change was calculated from the average normalized cycle difference of all biological repeats. Statistically significance (Benjamini-Hochberg FDR corrected p-value ≤ 0.1) is indicated with asterisks (see additional file 1).

To further study the role of SA, ET and JA in ROS signalling, wild type and the *sid2*, *npr1*, *dnd1*, *ein*2 and *fad3/7/8 *mutants were exposed to O_3_. A subset of 23 O_3_-induced and one O_3_-repressed *CRKs *were selected for expression analysis in the mutant backgrounds by qPCR (Figure 6). Most O_3_-induced CRKs exhibited even higher expression levels in *sid2 *and *npr1 *as compared to wild type, with the exception of *CRK10*, *CRK11*, *CRK20 *and *CRK29*. In *ein2*, the magnitude of *CRK *induction was reduced. In the JA-deficient *fad3/7/8 *mutant, the increased expression of *CRKs *in response to O_3 _was in several cases reduced or even absent as compared to wild type plants. Remarkably, O_3_-triggered increase in expression of *CRKs *was absent in *dnd1 *(Figure 6). In summary, these results suggest that the plant hormones SA, JA and ET play central roles in the regulation of the expression of the *CRK *subfamily, both under control conditions (clean air), as well as in response to O_3_.

**Figure 7:**
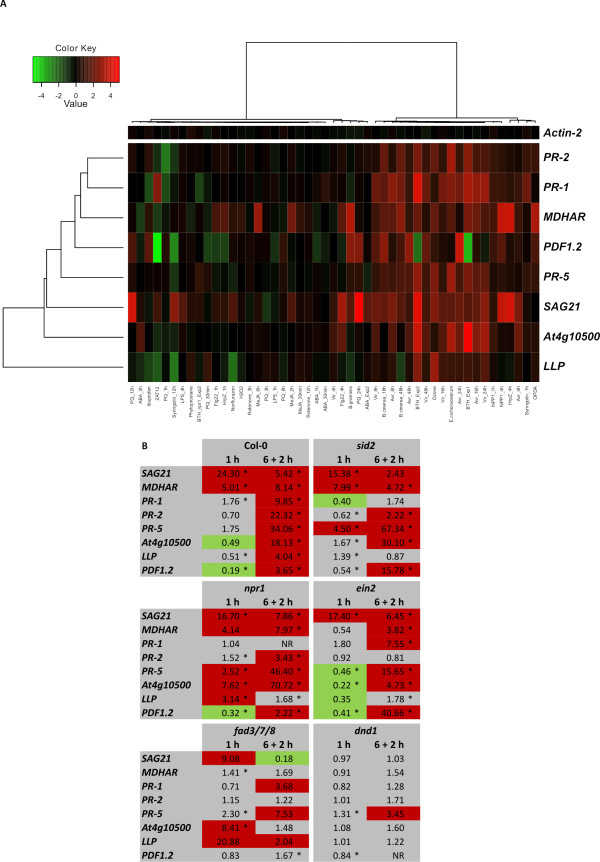
**Clustering and qPCR analysis of the marker genes**. (A) The expression of eight O_3_-inducible genes and the qPCR normalization gene *Actin-2 *were analyzed in public array data from biotic and abiotic stress and hormone treatments. Red and green indicate increased or decreased expression compared to untreated plants, respectively. The intensity of the colours is proportional to the absolute value of the fold difference. (B) Markers genes for O_3 _responses were analyzed by qPCR in Col-0 and *sid2*, *npr1*, *dnd1*, *ein2 *and *fad3*/7/8 exposed to 250 ppb O_3 _for 6 h. Samples were harvested at 1 or 8 h (6 h plus 2 h recovery under clean air conditions) after the onset of the O_3 _treatment. Transcript levels were calculated by comparison of O_3_-exposed plants with corresponding control plants grown under clean air conditions. An expression level of one indicates no change of expression, increased expression is indicated by values larger than one while decreased expression is shown by values smaller than one. Increase of expression by 2-fold or higher is high-lighted in red and decrease in expression by 2-fold or more in green. NR - no reproducible data could be obtained for this gene. The experiment was repeated four times; fold change was calculated from the average normalized cycle difference of all biological repeats. Statistically significance (Benjamini-Hochberg FDR corrected p-value ≤ 0.1) is indicated with asterisks (see additional file 1).

To expand the model for O_3 _regulated gene expression, we tested several other O_3 _inducible marker genes. These genes were selected to represent "classical" marker genes for SA (including *PATHOGENESIS-RELATED GENE 1 *[*PR-1*] and *PATHOGENESIS-RELATED GENE 2 *[*PR-2*] and JA/ET (*PLANT DEFENSIN 1.2 *[*PDF1.2*]). In addition we selected genes based on our previous O_3 _microarray data (*SENESCENCE-ASSOCIATED GENE 21 *[*SAG21*] [51]), and genes which have previously been described as JA-regulated (*MONODEHYDROASCORBATE REDUCTASE *[*MDHAR*] [52]) or SA- and NPR1-regulated (*LECTIN-LIKE PROTEIN *[*LLP*] *At5g03350 *[53]). The overall regulation of the marker genes was obtained by clustering them in response to biotic and abiotic stress and hormone treatments (Figure 7A). Most of the genes were regulated in response to BTH, biotic stress treatment and O_3_, and the *MDHAR *gene was confirmed as a JA marker gene, as previously reported [52]. However, there was a lack of overall "specificity" in marker gene expression, i.e., several hormones or stresses were altering their expression. The marker genes were next tested with qPCR in the same O_3 _samples used for *CRK *expression. The genes were strongly induced in Col-0 wild type plants and in most mutants. However, in *dnd1 *the O_3_-induced signalling pathway(s) was evidently blocked since O_3_-induced gene expression was not observed or it was severely reduced. Only *PATHOGENESIS-RELATED GENE 5 *(*PR-5*) was weakly induced in *dnd1 *at the later time point. The classical SA marker genes *PR-1 *and *PR-2 *had reduced O_3_-induced increased expression in *sid2 *and *npr1*, indicating a role for SA signalling in response to O_3_. The loss of O_3 _induction of *MDHAR *in *fad3/7/8 *confirmed the importance of JA in regulation of this gene.

### Light stress response of the CRKs in hormone mutants

**Figure 8:**
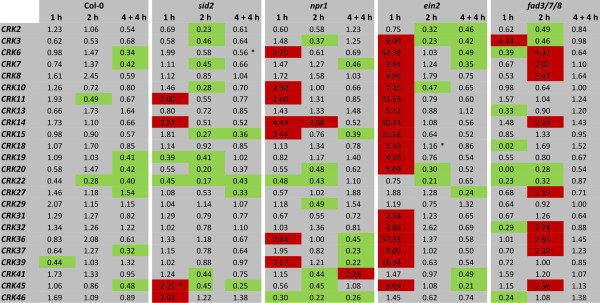
**Light stress response in hormone mutants**. The expression of 24 O_3_-inducible *CRKs *was analyzed by qPCR in Col-0 and *sid2*, *npr1*, *ein2 *and *fad3*/7/8 after 1 h and 2 h exposure to light stress conditions, and after 4 h light stress followed by 4 h recovery at normal growth light conditions. Transcript levels were calculated by comparison of light stress-treated plants with the corresponding control plants grown under normal light conditions. An expression level of one indicates no change of expression, increased expression is indicated by values larger than one while decreased expression is shown by values smaller than one. Increase in expression by 2-fold or higher is high-lighted in red and decrease in expression by 2-fold or more in green. NR - no reproducible data could be obtained for this gene. The experiment was repeated twice; fold change was calculated from the average normalized cycle difference of all biological repeats. Statistically significance (Benjamini-Hochberg FDR corrected p-value ≤ 0.1) is indicated with asterisks (see additional file 1).

To elucidate the role of SA, JA and ET in the regulation of *CRK *expression in response to light stress, wild type and the *sid2*, *npr1*, *ein2 *and *fad3/7/8 *mutants were exposed to light stress and the subset of O_3_-regulated *CRKs *was analyzed by qPCR. The transcriptional repression observed in response to light stress (Figure 3) for a majority of *CRK *family members was even more pronounced for some *CRKs *in *sid2 *(Figure 8). Interestingly, several *CRKs *were specifically transcriptionally induced by light stress in the *ein2 *mutant. In *fad3/7/8*, most *CRKs *exhibited a transient decrease in gene expression at early time points. However, statistical significance was overall low for the light-dependent regulation of the *CRKs *in the hormone signalling and biosynthesis mutants (Additional file 1).

### CRK promoter analysis

Gene expression is regulated by transcription factors and the promoter elements they bind to. The 500 base pair (bp) and 1000 bp upstream promoter regions of the *CRKs *were inspected for significantly enriched promoter elements based on a list of verified *Arabidopsis *promoter elements (http://arabidopsis.med.ohio-state.edu/AtcisDB/bindingsites.html[54]). The *CRKs *were divided into three groups ("*CRKs *all", "*CRKs *O_3 _up" - O_3 _increased expression and "*CRKs *O_3 _down" - O_3 _decreased expression) and searched for significant accumulation of single promoter elements or a combination of promoter elements. Statistical significance was measured with the Fisher exact test using false discovery rate correction [55]. The enrichment was calculated separately for the motifs in both forward and reverse orientations. No elements were enriched in the 1000 bp region for any of the groups or in the 500 bp region of O_3 _down genes. One element, the W-box, a target for WRKY transcription factors frequently found in the promoters of SA-regulated genes [56], was significantly overrepresented as a single motif in the group of "*CRKs *all" and "*CRKs *O_3 _up" in the 500 bp region (Table 2 and Additional file 3). Interestingly, several pairs of promoter elements were present with high statistical significance in the 500 bp region for the "*CRKs *O_3 _up" and "*CRKs *all" groups. Since these were mostly the same for both groups and had high statistical significance for the all group, this indicated that they were probably not responsible for the O_3_-regulation of these genes. The W-box was the only element enriched as a single motif but also present in most pairs of promoter elements. This indicated that the W-box, alone or in combination with other elements, could be a target for the SA and/or pathogen regulation of *CRKs*.

**Table 2:** Motifs overrepresented in the promoters of the CRK family.

Promoter motifs					
**Promoter motifs**					

**Number**	**Subset**	**Region**	**Motif**	**q-value**	**Motif name**

1	*CRKs *O_3 _up	500 bp	TTGAC(+)	0.05	W-box

2	*CRKs *all	500 bp	TTGAC(+/-)	0.05	W-box

3	*CRKs *all	500 bp	TTGAC(+)	0.01	W-box

4	*CRKs *all	500 bp	TTGAC(-)	0.01	W-box

5	*CRKs *O_3 _up up	500 bp	ACACNNG(+/-) × TTGAC(+)	0.00	DPBF1&2 × W-box

6	*CRKs *O_3 _up	500 bp	ACACNNG(-) × ACTTTG(+)	0.05	DPBF1&2 × T-box

7	*CRKs *O_3 _up	500 bp	ACACNNG(-) × TTGAC(+)	0.05	DPBF1&2 × W-box

8	*CRKs *O_3 _up	500 bp	A [AC]C [AT]A [AC]C(-) × TTGAC(+)	0.05	MYB4 × W-box

9	*CRKs *O_3 _up	500 bp	CAACA(-) × TTGAC(+)	0.05	RAV1-A × W-box

10	*CRKs *O_3 _up	500 bp	CAACA(-) × TTGAC(-)	0.05	RAV1-A × W-box

11	*CRKs *O_3 _up	500 bp	ACACNNG(-) × A [AC]C [AT]A [AC]C(-)	0.05	DPBF1&2 × MYB4

12	*CRKs *O_3 _up	500 bp	ACACNNG(-) × TTGAC(-)	0.05	DPBF1&2 × W-box

13	*CRKs *O_3 _up	500 bp	A [AC]C [AT]A [AC]C(-) × TTGAC(-)	0.05	MYB4 × W-box

14	*CRKs *all	500 bp	ACACNNG(+/-) × TTGAC(+)	0.03	DPBF1&2 × W-box

15	*CRKs *alll	500 bp	ACTTTG(+/-) × TTGAC(-)	0.04	T-box × W-box

16	*CRKs *all	500 bp	ACACNNG(-) × TTGAC(+)	0.05	DPBF1&2 × W-box

17	*CRKs *all	500 bp	GATAAG(-) × AAATTAGT(+)	0.05	Ibox × BS2

18	*CRKs *all	500 bp	CAACA(-) × TTGAC(+)	0.05	RAV1-A × W-box

19	*CRKs *all	500 bp	CAACA(-) × TTGAC(-)	0.01	RAV1-A × W-box

20	*CRKs *all	500 bp	ACACNNG(-) × TTGAC(-)	0.03	DPBF1&2 × W-box

21	*CRKs *all	500 bp	GATAAG(-) × ACTAATTT(-)	0.03	Ibox × BS3

22	*CRKs *all	500 bp	A [AC]C [AT]A [AC]C(-) × TTGAC(-)	0.03	MAB4 × W-box

The promoters of the CRK family were analyzed for enrichment of *Arabidopsis *verified promoter elements. Enrichment was calculated for single and double motifs in both plus and minus orientation. The CRKs were divided into three groups for the analysis: "*CRKs *all", "*CRKs *O_3 _up" - O_3 _increased expression and "*CRKs *O_3 _down" - O_3 _decreased expression. (+) motif on forward strand, (-) motif on reverse strand, (+/-) motif on either forward or reverse strand. The CRKs containing the respective motifs are shown in additional file

## Discussion

The RLK family is one of the largest gene families in the *Arabidopsis thaliana *genome. Several RLKs have previously been described to be involved in plant-microbe interactions [14,15,57-59] and abiotic stress [60,61]. Based on statistical analysis of gene expression data, RLKs in general, as well as the *CRK *subfamily, are more likely to have altered expression in response to abiotic and biotic stress than other *Arabidopsis *genes [40,62]. We analyzed the expression profile of the *CRKs *in detail using qPCR and array analysis under various stresses, growth conditions, and in different genetic backgrounds to obtain a better understanding of the signalling pathways leading to transcriptional regulation of the *CRKs *and to elucidate the role of apoplastic ROS in stress signalling.

The use of ROS as signalling molecules is a common feature of many stress responses [25]. Pathogen attack and perception of PAMPs are often associated with an oxidative burst in the apoplast [63]. Similarly, a hallmark of the early O_3 _response is the generation of an oxidative burst in the apoplast [64]. ROS are also produced in other subcellular compartments, including the chloroplast, where light stress or treatments with the herbicides paraquat or norflurazon elicit elevated ROS production. In addition, crosstalk between pathways elicited by apoplastic ROS and chloroplast-derived ROS is important for the regulation of cell death [32]. The transcriptional response to apoplastic ROS, e.g. induced by O_3_, is strikingly different from chloroplast-derived ROS, e.g., induced by paraquat [30]. To further dissect the role of apoplastic ROS, we clustered several treatments triggering ROS production in distinct subcellular compartments together with various biotic stress experiments. Our results showed that the *CRK *expression profile upon O_3 _exposure was most similar to those stimulated by PAMP perception (flg22 and HrpZ) and pathogen infection (*Bgh*) (Figure 4). By contrast, treatments, which increased ROS levels in the chloroplast (norflurazon and paraquat) or mitochondria (rotenone; which might also lead to ROS production in the chloroplast [65]) either had no effect on *CRK *gene expression or resulted in down-regulation. These results show that transcriptional induction of the *CRKs *can be triggered by apoplastic ROS, whereas chloroplastic ROS mainly lead to decreased expression. Furthermore, cluster analysis separated the effects of plant hormones: BTH (SA analog) caused a similar expression profile as O_3 _and PAMP treatments, whereas *CRK *expression in response to ABA and MeJA was related to norflurazon and paraquat treatments.

To extend the microarray meta-analysis, transcript accumulation of the *CRK *subfamily was monitored in response to O_3 _and light stress by qPCR. Out of 44 CRKs, 32 showed increased expression after exposure to O_3 _at both time points while five members exhibited decreased expression. Light stress treatment led to a decrease in expression of the majority of the *CRKs*. Thus, in agreement with the results from array analysis, ROS production in different cellular compartments produces strikingly different transcriptional profiles on the *CRK *gene subfamily.

To further dissect the O_3 _response, mutants deficient in biosynthesis, perception and signalling of SA (*sid2*, *npr1*), JA (*fad3/7/8*) and ET (*ein2*) were exposed to O_3 _and the expression of a subset of *CRKs *was analyzed by qPCR. The O_3_-induced increase in transcript levels of the *CRKs *was higher in *sid2 *and *npr1 *implying that SA acts as a negative regulator of the ROS signalling pathway. The O_3_-mediated transcriptional induction of *CRKs *was almost abolished in *fad3/7/8 *and attenuated in *ein2*, suggesting that JA, and to a lesser extent ET are required for the proper transcriptional induction of *CRKs *in response to O_3_. This role for SA, JA and ET in O_3 _signalling has been previously proposed based on the results from cDNA macroarray analysis [66]. The effect of light stress on the *CRK *expression in various mutant backgrounds was very different compared to the effect of the O_3 _response. Whereas ET acts as positive regulator of *CRK *expression in the O_3 _response, it appears to be a negative regulator in light stress since several *CRKs *displayed light stress-induced expression only in the *ein2 *mutant (Figure 8). Under light stress conditions, the decreased expression of *CRKs *seen in wild type was even more pronounced in the SA mutants *sid2 *and *npr1 *and the JA mutant *fad3/7/8*.

*DND1 *encodes CYCLIC NUCLEOTIDE GATED CHANNEL2 (CNGC2) which transports Ca^2+ ^into the cell and regulates nitric oxide production [67]. The complete lack of an effect of O_3 _on *CRK *and marker gene expression in *dnd1 *suggests an important role for CNGC2 in the O_3 _response pathway, possibly by regulating Ca^2+ ^levels (Figure 6 and 7B). Previous studies have shown that O_3 _rapidly invokes Ca^2+^ transients [68,69] and blocking of Ca^2+^ transport can prevent ROS-induced cell death [70]. The *dnd1 *mutant also has several pleiotropic phenotypes which include elevated SA levels and constitutive defence responses [47]. Consequently, the lack of O_3 _response in *dnd1 *could be due to "dominance" of SA signaling over the ROS signalling pathway, and O_3 _would have no effect when the SA pathway is fully stimulated. Previous reports have shown that several members of the *CRK *subfamily were transcriptionally induced through an external application of SA [19] or BTH (Figure 4). The response of *CRKs *to BTH was completely blocked in *npr1*, indicating that the SA pathway for regulating *CRKs *requires NPR1.

Intriguingly, different growth conditions had a strong impact on the expression of *CRKs *in various mutants. Several *CRKs *were expressed to higher levels in *ein2 *and *fad3/7/8 *in Weiss chamber-grown plants compared to Phytotron-grown plants. In contrast, the decreased expression of several *CRKs *in *sid2 *and *npr1 *was similar between two different growth conditions (Weiss chamber and Phytotron, Figure 5). A strong effect of environmental conditions on mutant phenotypes, transcript profiles and other parameters are well known and a common problem when comparing results from different laboratories [71]. There could be several reasons for the differences in the expression levels of the *CRKs *between the Weiss chambers and the Phytotron growth conditions. Plants were tested at slightly different ages and grown in different soil (see materials and methods section). Illumination in the Weiss chambers was provided using fluorescent lamps while in lighting in the Phytotron was using metal halide lamps with different light spectra. Notably, the *CRKs *are responsive to UV-B [40]. This suggests that light conditions could have an effect on the expression profile of this RLK family. Another reason for this variation of gene expression could be that under control conditions most *CRKs *were expressed at very low levels; consequently, a minor perturbation either by genetic mutation or growth condition could lead to altered expression. Thus, expression of *CRKs *is very sensitive to the surrounding environment. Similar observations have been reported for the expression of the classical *PDF1.2 *marker gene [49,50]. This gene has long been used to exemplify co-regulation by JA/ET. However, *PDF1.2 *is only regulated by both hormones when plants are grown *in vitro *[49]. When plants are grown in soil, either hormone alone (JA or ET) is sufficient to induce expression. Thus, growth in soil is able to induce or prime defence signalling pathways.

## Conclusions

**Figure 9:**
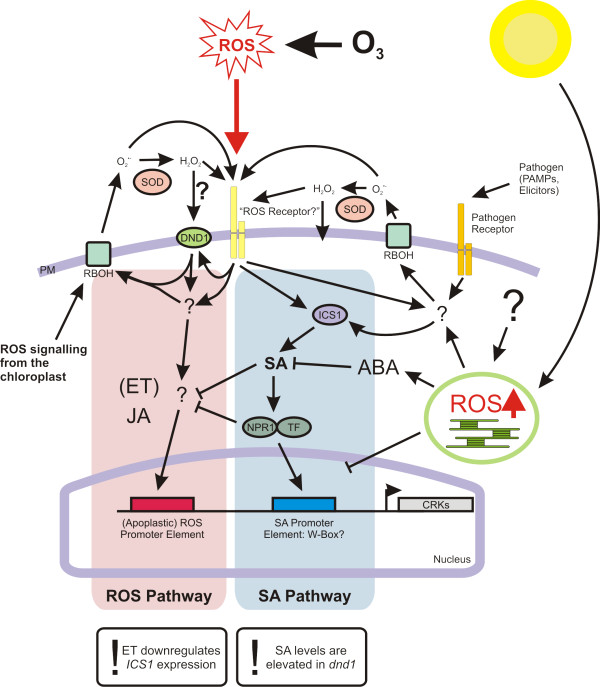
**ROS, elicitor and hormone regulation of O_3_-induced CRKs**. O_3 _enters the leaves through the stomata and immediately reacts with components of the cell wall to generate ROS. O_3 _and the ROS induce an active production of ROS in the apoplast which is at least partly depending on membrane bound NADPH oxidases (RBOH), which produce . Similar ROS production in the apoplast takes place after infection of a plant with a pathogen or treatments with pathogen derived elicitors (PAMPs). ROS is hypothetically perceived *via *a "ROS receptor" which could sense ROS directly *via *protein modification, or *via *sensing of modified apoplastic proteins or other molecules that react with ROS. The perception of ROS initiates down-stream signalling events. H_2_O_2 _is also able to cross the plasma membrane and enter the cells. Inside the cell, the signalling pathway is split into two pathways. In the ROS pathway DND1/CNGC2 mediates a required step of the signalling pathway and JA and ET act as positive regulators, and SA and NPR1 are negative regulators. In the SA pathway ROS or pathogens activate SA biosynthesis *via *ICS1; and NPR1 is a required component. Since NPR1 is a positive regulator of the SA pathway and a negative regulator of the ROS pathway this implies that the separate signalling pathway use different transcription factors and promoter elements to regulate *CRK *expression, although it might be possible that two different transcription factors could converge on the same promoter element. In addition the pleiotropic nature of the *dnd1 *mutant, including high SA-levels, could change the place of DND1/CNGC2 in the model - constitutive SA signalling in *dnd1 *may limit the possibility for O_3 _to activate the ROS pathway. Through the transcription factors EIN3 and EIL1 ET can repress SID2/ICS1 expression and SA levels. Increased ROS production in the chloroplast activates separate signalling pathway(s) leading to repression of *CRK *expression. One of these pathways could involve ABA and negative cross talk with the SA pathway.

Based on the *CRK *expression patterns and integrating current knowledge of ROS signalling, PAMP perception and light responses [25,26,38,72], we propose a model for the regulation of increased expression of the *CRKs *(Figure 9): O_3 _induces ROS production in the apoplast which is perceived by putative "ROS receptors" (or by other mechanisms) amplified by PLANT RESPIRATORY BURST OXIDASE HOMOLOG (RBOH)-mediated  production, thus leading to activation of DND1/CNGC2. This activates further down-stream signalling events where JA and to a lesser extent ET act as positive regulators, and SA and NPR1 as negative regulators of *CRK *expression. Eventually, the signal reaches the nucleus where transcription factors bind to a "ROS" promoter element and activate transcription. In parallel, the genes are also regulated through a SA (synthesized by ICS1) and NPR1-dependent pathway converging on the W-box promoter element. Microbes and PAMPs could activate both pathways at different timing; a rapid pathway would act through a RBOH mediated ROS production and use the "ROS pathway", while a later "SA pathway" requires increased SA biosynthesis and NPR1. Further interconnections between the pathways are provided by the primary ET transcription factors ETHYLENE INSENSITIVE 3 (EIN3) and ETHYLENE INSENSITIVE 3-LIKE (EIL1) which repress *SID2/ICS1 *expression and thus decrease SA levels [73]. Light stress or chemical treatments that increase ROS in the chloroplast activate separate signalling pathway(s) mainly leading to repression of *CRK *expression, which could involve ABA and negative crosstalk with the SA pathway.

Is it possible to separate the roles of chloroplastic and apoplastic ROS in the regulation of *CRK *expression? Chloroplast-derived ROS production is known to be involved in the regulation of cell death during pathogen infection and in response to abiotic stress [74,75]. Specific removal of chloroplastic ROS prevents pathogen-induced cell death but has no impact on defence gene expression [75]. Furthermore, chloroplastic ^1^O_2 _regulates cell death dependent on EXECUTER1 [31]. In comparison, apoplastic ROS might be involved with intra- and intercellular signalling [76]. Thus, apoplastic ROS would have a role in regulating defence gene expression and chloroplastic ROS in regulation of cell death. In addition, there is crosstalk between apoplastic ROS and chloroplast ROS; rapid ROS production in the chloroplast can be detected in response to O_3 _and blocking of ROS production in the chloroplast reduces O_3_-induced cell death [32,77]. Clearly, ROS regulation of defence signalling and/or cell death is very complex and several other regulatory components have been identified, including LESION SIMULATING DISEASE 1 (LSD1), ENHANCED DISEASE SUSCEPTIBILITY 1 (EDS1) and PHYTOALEXIN DEFICIENT 4 (PAD4), which are also involved in acclimation to light stress [42,78]. The only known phenotypes for CRKs have been obtained by ectopic overexpression, which induces HR-like cell death independent or dependent on SA signalling (depending on the specific CRK) [22,23]. How this induction of cell death might be achieved is still unclear since transcriptional regulation of *CRKs *occurs in response to apoplastic rather than chloroplastic ROS. Some members of the RLK family might participate in a positive feed-forward loop to regulate ROS production, defence gene expression, cell death and hormone signalling. This regulatory loop might be deregulated after overexpression of the CRKs leading to the observed cell death phenotypes. However, this will require experimental verification in the future.

What is the role of CRKs in plants and why are they regulated by PAMPs and O_3 _treatment? The external domain of these RLKs could be the receptor for as yet uncharacterized PAMPs and they could be part of plant immune responses. An intriguing feature of the DUF26 domain is the presence of a conserved cysteine motif C-8X-C-2X-C. The configuration of cysteines is similar to the cysteine motif in the GRIM REAPER protein, which has been shown to be involved in the regulation of ROS induced cell death [79]. Despite the ubiquitous role of ROS as signalling molecules in plants, no direct receptor for ROS has been described. Since cysteines are sensitive to redox modifications, could the DUF26 domain act as sensor of ROS in the apoplast and be the putative ROS sensor as depicted in Figure 9?

## Methods

### Plant growth conditions and treatments

#### Weiss chamber growth conditions

For exposure to O_3_, *Arabidopsis thaliana *Col-0 or mutant plants were grown in a peat/vermiculite (1:1) mixture for 21 days in Weiss 1300 growth cabinets (photon flux density 250 *μ*mol m^-2 ^sec^-1^; tubular fluorescent lamps) under 12 hours day length (day: 23°C 70% relative humidity; night 18°C 90% relative humidity). Lights were switched on at 7 AM and off at 7 PM. O_3 _treatments were started at 9 AM. 21-day old plants were used and exposed to 250 parts per billion (ppb) O_3 _for 6 hours. Samples were harvested at the times indicated in the respective experiments after the onset of the O_3 _treatment. Samples were taken in parallel from O_3 _treated and clean air control plants and immediately shock-frozen in liquid nitrogen.

#### Phytotron growth conditions

For light stress treatments, plants were grown on a pre-fertilized garden soil/vermiculite (1:1) mixture for 28 days under 8 h/16 h light/dark at 22 or 20°C, respectively, and 50% humidity at a light intensity of 130 *μ*mol m^-2 ^sec^-1 ^photon flux density (Metal halide lamps). For light stress treatment, plants were shifted to 1300 *μ*mol m^-2 ^sec^-1 ^photon flux density for up to 4 hours. Subsequently, plants were returned to a light intensity of 130 *μ*mol m^-2 ^sec^-1 ^photons. Controls were kept at 130 *μ*mol photon flux density throughout the duration of the treatment and samples were taken in parallel with the light stress-treated plants. Samples were harvested at the times indicated in the respective experiments after the onset of the light stress treatment and immediately shock-frozen in liquid nitrogen.

For flg22 treatments, plants were grown on MS plates with Nitsch vitamins (MSN). After 7 days, seedlings were transferred to liquid MSN media and cultivated for 7 days. Before the flg22 treatment, fresh medium was added. After a 1 hour recovery period, the seedlings were treated with 100 nM flg22. Controls were treated with H_2_O. Samples were harvested at the times indicated in the respective experiments after the onset of the treatment and in parallel from corresponding controls and immediately shock-frozen in liquid nitrogen.

### RNA extraction and qPCR analysis

RNA was isolated as described [79]. 5 *μ*g total RNA was DNaseI treated (Fermentas) and used for cDNA synthesis with RevertAid Premium Reverse Transcriptase (Fermentas) and Ribolock RNase Inhibitor (Fermentas) according to manufacturers' instructions. The reaction was diluted to a final volume of 50 *μ*l and 1 *μ*l cDNA was used as template for PCR using LightCycler 480 SYBR Green I master mix (Roche Diagnostics) on a LightCycler 480 (Roche Diagnostics) in triplicate. Primer sequences and the primer amplification efficiency (E_*x*_; determined according to manufacturers instructions) are available in additional file 4.

For the normalization of the data several genes were evaluated to select a suitable gene for normalization based on the method of Vandesompele *et al*. [80]. *Actin-2 *(*At3g18780*) was found to be stably expressed in control and ozone treated plants and was subsequently used for normalization. The raw Ct values were normalized to *Actin-2 *and used to compare the results from untreated control samples with treated samples using the 2^-ΔΔ*Ct *^method. The resulting normalized cycle differences were used to calculate the average (*μ*) and standard deviation (*σ*) of the biological repeats and the p-value (using SPSS) based on [81]. The p-value was calculated using the one-sample t-test in SPSS and calibrated using the Benjamini-Hochberg false discovery rate (FDR) correction [82]. The 95% confidence intervals (CI_±_; lower and upper bound) were calculated according to , where E_*x *_is the efficiency of the reaction *x*. The *μ*, *σ*, CI and p-value for all qPCR experiments are shown in additional file 1. The mean *μ *of the normalized cycle difference was used to calculate the fold-change of expression using E_*x *_(Additional file 4).

### Phylogenetic analysis

RLK kinase domains were identified using PrositeScan http://au.expasy.org/tools/scanprosite/. Sequence alignments were performed using the ClustalW2 program [83]. Neighbour-joining trees were constructed with 1000 bootstrap sets using the Mega4 software package [84].

### Micro-array analysis

Affymetrix raw data was downloaded from NASCArrays http://affymetrix.arabidopsis.info/narrays/experimentbrowse.pl (accession number NASCARRAYS-143, paraquat; NASCARRAYS-353, ZAT12; NASCARRAYS-176, ABA time course experiment 1; NASCARRAYS-192, Ibuprofen), ArrayExpress http://www.ebi.ac.uk/microarray-as/ae/(accession numbers E-GEOD-12856, *Blumeria graminis *sp. *hordei*; E-GEOD-5684, *Botrytis cinerea*; E-ATMX-13, Methyl Jasmonate; E-MEXP-739, Syringolin A; E-MEXP-1797, Rotenone), Gene Expression Omnibus http://www.ncbi.nlm.nih.gov/geo/(accession numbers GSE5615, Elicitors LPS, HrpZ, Flg22 and NPP1; GSE5685, Virulent and avirulent *Pseudomonas syringae*:; GSE9955, BTH experiment 1, GDS417 *E. cichoracearum*; GSE5530, H_2_O_2_; GSE5722, O_3_; GSE12887, Norflurazon; GSE10732, OPDA and Phytoprostane; GSE7112, ABA experiment 2) and The Integrated Microarray Database System http://ausubellab.mgh.harvard.edu/imds (Experiment name: BTH time course, BTH experiment 2).

The raw Affymetrix data was preprocessed with RMA using probe set annotations (custom cdf files) from http://brainarray.mbni.med.umich.edu/, version 11.0.1. Biological repeats of each experiment were combined by computing a mean of the measured gene expression. Gene expression was summarized by computing a log2 ratio of the treatment and control expressions (differential expression, DE). A visualization of the DE values is shown in Figure 4. Variation of differential expression in an experiment e, , was estimated by summing the variances of (logarithm of) treatment and control gene expressions.

Parametric bootstrapping was implemented by generating 1000 samples for each experiment and each gene from a Gaussian distribution with the estimated DE as the mean and  as the variance.

Bootstrap samples were discretized to down regulated (log2 DE < -1), no regulation (-1 < log2 DE < 1), and up regulated (log2 DE > 1) genes. Bayesian agglomerative hierarchical clustering algorithm was then applied to the discretized bootstrap data. The Bayesian hierarchical clustering algorithm computes the best number of clusters by Bayesian hypothesis testing. For each pair of genes (and experiments, depending on the clustering direction), the number of times they were assigned to the same cluster was computed. These gene (or experiment) similarities were then used as distances for computing the hierarchical clustering (ward method) shown in Figure 4.

### Promoter analysis

TAIR 9 version of promoter sequences of 500 bases and 1000 bases upstream of the *Arabidopsis *genes was downloaded from http://www.arabidopsis.org/. A list of verified *Arabidopsis *promoter elements was taken from http://arabidopsis.med.ohio-state.edu/AtcisDB/bindingsites.html[54]. The set of *CRKs *was divided into three groups (all, ozone up-regulated and ozone down-regulated) and the plus and minus strands of the promoters were searched for significant enrichment of single promoter elements or a combination of two promoter elements in either of the strands. Fisher exact test with false discovery rate correction (q-values; [55]) was used for measuring the significance of the enrichment; q-value of 0.05 was used as the threshold.

## Authors' contributions

MW, MB, SR, SK, BK and JK designed research. MW, MB, JS, NI, SLK and SM carried out research. MW, MB, JS and JK analyzed the data. MW, MB and JK wrote the paper. All authors have read and approved the final manuscript.

## Supplementary Material

Additional file 1 **Lower and upper percentiles and p-values**. The raw normalized cycle differences (ΔΔ*Ct*) for all experiments, their average, standard deviation, geometric mean, lower and upper percentile and the Benjamini-Hochberg False Discovery Rate-corrected p-value for all experiments is shown in the Excel File. Each Excel worksheet represents data for a Figure showing qPCR data. Click here for file

Additional file 2 **Transcriptional regulation of the CRKs in response to flg22**. 14-day old *Arabidopsis *Col-0 were treated with 100 nM flg22 and samples taken after 30 and 60 minutes (water-treated control samples have been harvested at the same time points in parallel). Expression of several *CRKs *was analyzed by qPCR. Transcript levels were calculated by comparison with the corresponding control plants. An expression level of one indicates no change in expression, increased expression is indicated by values larger than one while decreased expression is shown by values smaller than one. Increase in expression by 2-fold or higher is high-lighted in red and decrease in expression by 2-fold or more in green. Click here for file

Additional file 3 **List of CRKs for promoter motifs in table 2**. This file lists the AGI codes for the CRKs containing the promoter motif combinations shown in table 2. Click here for file

Additional file 4 **Primer sequences for qPCR analysis**. All primer sequences used for qPCR analysis in the manuscript plus the experimentally determined primer amplification efficiencies E_*x *_are listed. Click here for file

## References

[B1] Shiu SH Bleecker AB Expansion of the receptor-like kinase/Pelle gene family and receptor-like proteins in Arabidopsis Plant Physiol 2003 132 2 530 43 10.1104/pp.103.021964 12805585 PMC166995

[B2] Shiu SH Karlowski WM Pan R Tzeng YH Mayer KFX Li WH Comparative analysis of the receptor-like kinase family in Arabidopsis and rice Plant Cell 2004 16 5 1220 34 10.1105/tpc.020834 15105442 PMC423211

[B3] Shiu SH Bleecker AB Receptor-like kinases from *Arabidopsis *form a monophyletic gene family related to animal receptor kinases Proc Natl Acad Sci USA 2001 98 19 10763 8 10.1073/pnas.181141598 11526204 PMC58549

[B4] Shiu SH Bleecker AB Plant receptor-like kinase gene family: diversity, function, and signaling Sci STKE 2001 2001 113 RE22 10.1126/stke.2001.113.re22 11752632

[B5] Ogawa M Shinohara H Sakagami Y Matsubayashi Y *Arabidopsis *CLV3 peptide directly binds CLV1 ectodomain Science 2008 319 5861 294 10.1126/science.1150083 18202283

[B6] Escobar-Restrepo JM Huck N Kessler S Gagliardini V Gheyselinck J Yang WC Grossniklaus U The FERONIA receptor-like kinase mediates male-female interactions during pollen tube reception Science 2007 317 5838 656 60 10.1126/science.1143562 17673660

[B7] Stein JC Howlett B Boyes DC Nasrallah ME Nasrallah JB Molecular cloning of a putative receptor protein kinase gene encoded at the self-incompatibility locus of *Brassica oleracea* Proc Natl Acad Sci USA 1991 88 19 8816 20 10.1073/pnas.88.19.8816 1681543 PMC52601

[B8] Stein JC Dixit R Nasrallah ME Nasrallah JB SRK, the stigma-specific S locus receptor kinase of Brassica, is targeted to the plasma membrane in transgenic tobacco Plant Cell 1996 8 3 429 45 10.1105/tpc.8.3.429 8721749 PMC161111

[B9] van Zanten M Snoek LB Proveniers MCG Peeters AJM The many functions of ERECTA Trends Plant Sci 2009 14 4 214 8 10.1016/j.tplants.2009.01.010 19303350

[B10] Li J Wen J Lease KA Doke JT Tax FE Walker JC BAK1, an *Arabidopsis *LRR receptor-like protein kinase, interacts with BRI1 and modulates brassinosteroid signaling Cell 2002 110 2 213 22 10.1016/S0092-8674(02)00812-7 12150929

[B11] Nam KH Li J BRI1/BAK1, a receptor kinase pair mediating brassinosteroid signaling Cell 2002 110 2 203 12 10.1016/S0092-8674(02)00814-0 12150928

[B12] Chinchilla D Zipfel C Robatzek S Kemmerling B Nürnberger T Jones JDG Felix G Boller T A flagellin-induced complex of the receptor FLS2 and BAK1 initiates plant defence Nature 2007 448 7152 497 500 10.1038/nature05999 17625569

[B13] Heese A Hann DR Gimenez-Ibanez S Jones AME He K Li J Schroeder JI Peck SC Rathjen JP The receptor-like kinase SERK3/BAK1 is a central regulator of innate immunity in plants Proc Natl Acad Sci USA 2007 104 29 12217 22 10.1073/pnas.0705306104 17626179 PMC1924592

[B14] Zipfel C Kunze G Chinchilla D Caniard A Jones JDG Boller T Felix G Perception of the bacterial PAMP EF-Tu by the receptor EFR restricts *Agrobacterium*-mediated transformation Cell 2006 125 4 749 60 10.1016/j.cell.2006.03.037 16713565

[B15] Song WY Wang GL Chen LL Kim HS Pi LY Holsten T Gardner J Wang B Zhai WX Zhu LH Fauquet C Ronald P A receptor kinase-like protein encoded by the rice disease resistance gene, *Xa21* Science 1995 270 5243 1804 6 10.1126/science.270.5243.1804 8525370

[B16] Wang GL Ruan DL Song WY Sideris S Chen L Pi LY Zhang S Zhang Z Fauquet C Gaut BS Whalen MC Ronald PC *Xa21D *encodes a receptor-like molecule with a leucine-rich repeat domain that determines race-specific recognition and is subject to adaptive evolution Plant Cell 1998 10 5 765 79 10.1105/tpc.10.5.765 9596635 PMC144027

[B17] Xu WH Wang YS Liu GZ Chen X Tinjuangjun P Pi LY Song WY The autophosphorylated Ser686, Thr688, and Ser689 residues in the intracellular juxtamembrane domain of XA21 are implicated in stability control of rice receptor-like kinase Plant J 2006 45 5 740 51 10.1111/j.1365-313X.2005.02638.x 16460508

[B18] Lee SW Han SW Sririyanum M Park CJ Seo YS Ronald PC A type I-secreted, sulfated peptide triggers XA21-mediated innate immunity Science 2009 326 5954 850 24 10.1126/science.1173438 19892983

[B19] Chen K Fan B Du L Chen Z Activation of hypersensitive cell death by pathogen-induced receptor-like protein kinases from *Arabidopsis* Plant Mol Biol 2004 56 2 271 83 10.1007/s11103-004-3381-2 15604743

[B20] Czernic P Visser B Sun W Savouré A Deslandes L Marco Y Van Montagu M Verbruggen N Characterization of an *Arabidopsis thaliana *receptor-like protein kinase gene activated by oxidative stress and pathogen attack Plant J 1999 18 3 321 7 10.1046/j.1365-313X.1999.00447.x 10377997

[B21] Chen Z A superfamily of proteins with novel cysteine-rich repeats Plant Physiol 2001 126 2 473 6 10.1104/pp.126.2.473 11402176 PMC1540112

[B22] Chen K Du L Chen Z Sensitation of defense responses and activation of programmed cell death by a pathogen-induced receptor-like protein kinase in *Arabidopsis* Plant Mol Biol 2003 53 61 74 10.1023/B:PLAN.0000009265.72567.58 14756307

[B23] Acharya BR Raina S Maqbool SB Jagadeeswaran G Mosher SL Appel HM Schultz JC Klessig DF Raina R Overexpression of CRK13, an Arabidopsis cysteine-rich receptor-like kinase, results in enhanced resistance to *Pseudomonas syringae* Plant J 2007 50 3 488 99 10.1111/j.1365-313X.2007.03064.x 17419849

[B24] Overmyer K Brosché M Kangasjärvi J Reactive oxygen species and hormonal control of cell death Trends Plant Sci 2003 8 7 335 42 10.1016/S1360-1385(03)00135-3 12878018

[B25] Apel K Hirt H Reactive oxygen species: metabolism, oxidative stress, and signal transduction Annu Rev Plant Biol 2004 55 373 99 10.1146/annurev.arplant.55.031903.141701 15377225

[B26] Miller G Shulaev V Mittler R Reactive oxygen signaling and abiotic stress Physiol Plant 2008 133 3 481 9 10.1111/j.1399-3054.2008.01090.x 18346071

[B27] Mehler AH Studies on reactions of illuminated chloroplasts. I. Mechanism of the reduction of oxygen and other Hill reagents Arch Biochem 1951 33 65 77 10.1016/0003-9861(51)90082-3 14857775

[B28] Bartoli CG Pastori GM Foyer CH Ascorbate biosynthesis in mitochondria is linked to the electron transport chain between complexes III and IV Plant Physiol 2000 123 335 44 10.1104/pp.123.1.335 10806250 PMC59007

[B29] Bechtold U Richard O Zamboni A Gapper C Geisler M Pogson B Karpinski S Mullineaux PM Impact of chloroplastic- and extracellular-sourced ROS on high light-responsive gene expression in *Arabidopsis* J Exp Bot 2008 59 2 121 33 10.1093/jxb/erm289 18212028

[B30] Gadjev I Vanderauwera S Gechev TS Laloi C Minkov IN Shulaev V Apel K Inzé D Mittler R Van Breusegem F Transcriptomic footprints disclose specificity of reactive oxygen species signaling in Arabidopsis Plant Physiol 2006 141 2 436 45 10.1104/pp.106.078717 16603662 PMC1475436

[B31] Kim C Meskauskiene R Apel K Laloi C No single way to understand singlet oxygen signalling in plants EMBO Rep 2008 9 5 435 9 10.1038/embor.2008.57 18451767 PMC2373375

[B32] Joo JH Wang S Chen JG Jones AM Fedoroff NV Different signaling and cell death roles of heterotrimeric G protein *α *and *β *subunits in the Arabidopsis oxidative stress response to ozone Plant Cell 2005 17 3 957 70 10.1105/tpc.104.029603 15705948 PMC1069711

[B33] Santner A Estelle M Recent advances and emerging trends in plant hormone signalling Nature 2009 459 7250 1071 8 10.1038/nature08122 19553990

[B34] Grant MR Jones JDG Hormone (dis)harmony moulds plant health and disease Science 2009 324 5928 750 52 10.1126/science.1173771 19423816

[B35] Adie BAT Pérez-Pérez J Pérez-Pérez MM Godoy M Sánchez-Serrano JJ Schmelz EA Solano R ABA is an essential signal for plant resistance to pathogens affecting JA biosynthesis and the activation of defenses in *Arabidopsis* Plant Cell 2007 19 5 1665 81 10.1105/tpc.106.048041 17513501 PMC1913739

[B36] Fan J Hill L Crooks C Doerner P Lamb C Abscisic acid has a key role in modulating diverse plant-pathogen interactions Plant Physiol 2009 150 4 1750 61 10.1104/pp.109.137943 19571312 PMC2719142

[B37] Ton J Flors V Mauch-Mani B The multifaceted role of ABA in disease resistance Trends Plant Sci 2009 14 6 310 7 10.1016/j.tplants.2009.03.006 19443266

[B38] Galvez-Valdivieso G Fryer MJ Lawson T Slattery K Truman W Smirnoff N Asami T Davies WJ Jones AM Baker NR Mullineaux PM The high light response in *Arabidopsis *involves ABA signaling between vascular and bundle sheath cells Plant Cell 2009 21 7 2143 62 10.1105/tpc.108.061507 19638476 PMC2729609

[B39] Overmyer K Tuominen H Kettunen R Betz C Langebartels C Sandermann H Kangasjärvi J Ozone-sensitive Arabidopsis *rcd1 *mutant reveals opposite roles for ethylene and jasmonate signaling pathways in regulating superoxide-dependent cell death Plant Cell 2000 12 10 1849 62 10.1105/tpc.12.10.1849 11041881 PMC149124

[B40] Lehti-Shiu MD Zou C Hanada K Shiu SH Evolutionary history and stress regulation of plant receptor-like kinase/pelle genes Plant Physiol 2009 150 12 26 10.1104/pp.108.134353 19321712 PMC2675737

[B41] Swarbreck D Wilks C Lamesch P Beradrini TZ Garcia-Hernandez M Foerster H Li D Meyer T Muller R Ploetz L Radenbaugh A Singh S Swing V Tissier C Zhang P Huala E The Arabidopsis Information Resource (TAIR): gene structure and function annotation Nucleic Acids Res 2008 36 D1009 D1014 10.1093/nar/gkm965 17986450 PMC2238962

[B42] Mühlenbock P Szechynska-Hebda M Plaszczyca M Baudo M Mateo A Mullineaux PM Parker JE Karpinska B Karpinski S Chloroplast signaling and *LESION SIMULATING DISEASE1 *regulate crosstalk between light acclimation and immunity in *Arabidopsis* Plant Cell 2008 20 9 2339 56 10.1105/tpc.108.059618 18790826 PMC2570729

[B43] Savage RS Heller K Xu Y Ghahramani Z Truman WM Grant M Denby KJ Wild DL R/BHC: fast Bayesian hierarchical clustering for microarray data BMC Bioinformatics 2009 10 242 10.1186/1471-2105-10-242 19660130 PMC2736174

[B44] Bhattacharjee A Richards WG Staunton J Li C Monti S Vasa P Ladd C Beheshti J Bueno R Gillette M Loda M Weber G Mark EJ Lander ES Wong W Johnson BE Golub TR Sugarbaker DJ Meyerson M Classification of human lung carcinomas by mRNA expression profiling reveals distinct adenocarcinoma subclasses Proc Natl Acad Sci USA 2001 98 24 13790 5 10.1073/pnas.191502998 11707567 PMC61120

[B45] Clough SJ Fengler KA Yu IC Lippok B Smith RK Bent AF The *Arabidopsis dnd1 *"defense, no death" gene encodes a mutated cyclic nucleotide-gated ion channel Proc Natl Acad Sci USA 2000 97 16 9323 8 10.1073/pnas.150005697 10900264 PMC16866

[B46] Jones JDG Dangl JL The plant immune system Nature 2006 444 7117 323 9 10.1038/nature05286 17108957

[B47] Yu IC Parker J Bent AF Gene-for-gene disease resistance without the hypersensitive response in *Arabidopsis dnd1 *mutant Proc Natl Acad Sci USA 1998 95 13 7819 24 10.1073/pnas.95.13.7819 9636234 PMC22769

[B48] Genger RK Jurkowski GI McDowell JM Lu H Jung HW Greenberg JT Bent AF Signaling pathways that regulate the enhanced disease resistance of *Arabidopsis *"*Defense, No Death*" mutants Plant Mol Biol 2008 21 10 1285 96 10.1094/MPMI-21-10-1285 PMC2923831 18785824

[B49] Penninckx IAMA Thomma BPHJ Buchala A Métraux JP Broekaert WF Concomitant activation of jasmonate and ethylene response pathways is required for induction of a plant defensin gene in Arabidopsis Plant Cell 1998 10 12 2103 113 10.1105/tpc.10.12.2103 9836748 PMC143966

[B50] Mang HG Laluk KA Parsons EP Kosma DK Cooper BR Park HC AbuQamar S Boccongelli C Miyazaki S Consiglio F Chilosi G Bohnert HJ Bressan RA Mengiste T Jenks MA The Arabidopsis *RESURRECTION1 *gene regulates a novel antagonistic interaction in plant defense to biotrophs and necrotrophs Plant Physiol 2009 151 290 305 10.1104/pp.109.142158 19625635 PMC2735982

[B51] Ahlfors R Brosché M Kollist H Kangasjärvi J Nitric oxide modulates ozone-induced cell death hormone biosynthesis and gene expression in *Arabidopsis thaliana* Plant J 2009 58 1 12 10.1111/j.1365-313X.2008.03756.x 19054359

[B52] Sasaki-Sekimoto Y Taki N Obayashi T Aono M Matsumoto F Sakurai N Suzuki H Hirai MY Noji M Saito K Masuda T Takamiya K Shibata D Ohta H Coordinated activation of metabolic pathways for antioxidants and defence compounds by jasmonates and their roles in stress tolerance in Arabidopsis Plant J 2005 44 4 653 68 10.1111/j.1365-313X.2005.02560.x 16262714

[B53] Blanco F Salinas P Cecchini N Jordana X Hummelen PV Alvarez ME Holuigui L Early genomic responses to salicylic acid in Arabidopsis Plant Mol Biol 2009 70 1-2 79 102 10.1007/s11103-009-9458-1 19199050

[B54] Palaniswamy SK James S Sun H Lamb RS Davuluri RV Grotewold E AGRIS and AtRegNet. a platform to link cis-regulatory elements and transcription factors into regulatory networks Plant Physiol 2006 140 3 818 29 10.1104/pp.105.072280 16524982 PMC1400579

[B55] Storey JD A direct approach to false discovery rates Journal of the Royal Statistical Society: Series B (Statistical Methodology) 2002 64 3 479 98 10.1111/1467-9868.00346

[B56] Dong J Chen C Chen Z Expression profiles of the *Arabidopsis *WRKY gene superfamily during plant defense response Plant Mol Biol 2003 51 21 37 10.1023/A:1020780022549 12602888

[B57] Gómez-Gómez L Boller T FLS2: an LRR receptor-like kinase involved in the perception of the bacterial elicitor flagellin in *Arabidopsis* Mol Cell 2000 5 6 1003 11 10.1016/S1097-2765(00)80265-8 10911994

[B58] Sun X Cao Y Yang Z Xu C Li X Wang S Zhang Q *Xa26*, a gene conferring resistance to *Xanthomonas oryzae *pv. *oryzae *in rice, encodes an LRR receptor kinase-like protein Plant J 2004 37 4 517 27 10.1046/j.1365-313X.2003.01976.x 14756760

[B59] Zipfel C Robatzek S Navarro L Oakeley EJ Jones JDG Felix G Boller T Bacterial disease resistance in *Arabidopsis *through flagellin perception Nature 2004 428 6984 764 7 10.1038/nature02485 15085136

[B60] Sivaguru M Ezaki B He ZH Tong H Osawa H Baluska F Volkmann D Matsumoto H Aluminum-induced gene expression and protein localization of a cell wall-associated receptor kinase in Arabidopsis Plant Physiol 2003 132 4 2256 66 10.1104/pp.103.022129 12913180 PMC181309

[B61] Osakabe Y Maruyama K Seki M Satou M Shinozaki K Yamaguchi-Shinozaki K Leucine-rich repeat receptor-like kinase1 is a key membrane-bound regulator of abscisic acid early signaling in Arabidopsis Plant Cell 2005 17 4 1105 19 10.1105/tpc.104.027474 15772289 PMC1087989

[B62] Chae L Sudat S Dudoit S Zhu T Luan S Diverse transcriptional programs associated with environmental stress and hormones in the *Arabidopsis *Receptor-Like Kinase gene family Mol Plant 2009 2 84 107 10.1093/mp/ssn083 19529822 PMC2639733

[B63] Grant JJ Loake GJ Role of reactive oxygen intermediates and cognate redox signaling in disease resistance Plant Physiol 2000 124 21 9 10.1104/pp.124.1.21 10982418 PMC1539275

[B64] Langebartels C Ernst D Kangasjärvi J Sandermann H Ozone effects on plant defense Methods Enzymol 2000 319 520 35 full_text 10907540 10.1016/s0076-6879(00)19049-4

[B65] Ivanov B Asada K Edwards GE Analysis of donors of electrons to photosystem I and cyclic electron flow be redox kinetics of P700 in chloroplasts isolated bundle sheath strands of maize Photosynth Res 2007 92 65 74 10.1007/s11120-007-9166-0 17551845

[B66] Tamaoki M Nakajima N Kubo A Aono M Matsuyama T Saji H Transcriptome analysis of O_3_-exposed Arabidopsis reveals that multiple signal pathways act mutually antagonistically to induce gene expression Plant Mol Biol 2003 53 4 443 56 10.1023/B:PLAN.0000019064.55734.52 15010611

[B67] Ali R Ma W Lemtiri-Chlieh F Tsaltas D Leng Q von Bodman S Berkowitz GA Death don't have no mercy and neither does calcium: *Arabidopsis *CYCLIC NUCLEOTIDE GATED CHANNEL2 and innate immunity Plant Cell 2007 19 3 1081 95 10.1105/tpc.106.045096 17384171 PMC1867353

[B68] Evans NH McAinsh MR Hetherington AM Knight MR ROS perception in *Arabidopsis thaliana*: the ozone-induced calcium response Plant J 2005 41 4 615 26 10.1111/j.1365-313X.2004.02325.x 15686524

[B69] Ma W Smiegel A Verma R Berkowitz GA Cyclic nucleotidegated channels and related signaling components in plant innate immunity Plant Signal Behav 2009 4 4 272 82 10.4161/psb.4.4.8103 PMC2664486 19794842

[B70] Overmyer K Brosché M Pellinen R Kuittinen T Tuominen H Ahlfors R Keinänen M Saarma M Scheel D Kangasjärvi J Ozone-induced programmed cell death in the Arabidopsis *radical-induced cell death1 *mutant Plant Physiol 2005 137 3 1092 104 10.1104/pp.104.055681 15728341 PMC1065409

[B71] Carrera J Rodrigo G Jaramillo A Elena S Reverse-engineering *Arabidopsis thaliana *transcriptional network under changing environmental conditions Genome Biol 2009 10 9 R96 10.1186/gb-2009-10-9-r96 19754933 PMC2768985

[B72] Zipfel C Early molecular events in PAMP-triggered immunity Curr Opin Plant Biol 2009 12 4 414 20 10.1016/j.pbi.2009.06.003 19608450

[B73] Chen H Xue L Chintamanani S Germain H Lin H Cui H Cai R Zuo J Tang X Li X Guo H Zhou JM ETHYLENE INSENSITIVE3 and ETHYLENE INSENSITIVE3-LIKE1 repress *SALICYLIC ACID INDUCTION DEFICIENT2 *expression to negatively regulate plant innate immunity in *Arabidopsis* Plant Cell 2009 21 8 2527 40 10.1105/tpc.108.065193 19717619 PMC2751940

[B74] Liu Y Ren D Pike S Pallardy S Gassmann W Zhang S Chloroplast-generated reactive oxygen species are involved in hypersensitive response-like cell death mediated by a mitogen-activated protein kinase cascade Plant J 2007 51 6 941 54 10.1111/j.1365-313X.2007.03191.x 17651371

[B75] Zurbriggen MD Carrillo N Tognetti VB Melzer M Peisker M Hause B Hajirezaei MR Chloroplast-generated reactive oxygen species play a major role in localized cell death during the non-host interaction between tobacco and *Xanthomonas campestris *pv. *vesicatoria* Plant J 2009 60 962 73 10.1111/j.1365-313X.2009.04010.x 19719480

[B76] Miller G Schlauch K Tam R Cortes D Torres MA Shulaev V Dangl JL Mittler R The plant NADPH oxidase RBOHD mediates rapid systemic signaling in response to diverse stimuli Sci Signal 2009 2 84 ra45 10.1126/scisignal.2000448 19690331

[B77] Vahisalu T Puzõrjova I Brosché M Valk E Lepiku M Moldau H Pechter P Wang YS Lindgreen O Salojärvi J Loog M Kangasjärvi J Kollist H Ozone-triggered rapid stomatal response involves production of reactive oxygen species and is controlled by SLAC1 and OST1 Plant J 2010 62 442 453 Accepted article doi: 10.1111/j.1365-313X.2010.04159.x 20128877 10.1111/j.1365-313X.2010.04159.x

[B78] Mateo A Mühlenbock P Rustérucci C Chang CCC Miszalski Z Karpinska B Parker JE Mullineaux PM Karpinski S *LESION SIMULATING DISEASE 1 *is required for acclimation to conditions that promote excess excitation energy Plant Physiol 2004 136 2818 30 10.1104/pp.104.043646 15347794 PMC523344

[B79] Wrzaczek M Brosché M Kollist H Kangasjärvi J *Arabidopsis *GRI is involved in the regulation of cell death induced by extracellular ROS Proc Natl Acad Sci USA 2009 106 13 5412 7 10.1073/pnas.0808980106 19279211 PMC2664059

[B80] Vandesompele J Preter KD Pattyn F Poppe B Roy NV Paepe AD Spelemann F Accurate normalization of real-time quantitative RT-PCR data by geometric averaging of multiple internal control genes Genome Biol 2002 3 7 0034.1 0034.11 10.1186/gb-2002-3-7-research0034 PMC126239 12184808

[B81] Applied-Biosystems Relative quantitation of gene expression: ABI PRISM 7700 Sequence Detection System. User bulletin #2: Rev. B Weiterstadt, Germany, Applied Biosystems 2001

[B82] Benjamini Y Hochberg Y Controlling the false discovery rate: a practical and powerful approach to multiple testing J Royal Stat Soc, Series B 1995 57 289 300

[B83] Larkin MA Blackshields G Brown NP Chenna R McGettigan PA McWilliam H Valentin F Wallace IM Wilm A Lopez R Thompson JD Gibson TJ Higgins DG Clustal W and Clustal X version 2.0 Bioinformatics 2007 23 21 2947 8 10.1093/bioinformatics/btm404 17846036

[B84] Tamura K Dudley J Nei M Kumar S MEGA4: Molecular Evolutionary Genetics Analysis (MEGA) software version 4.0 Mol Biol Evol 2007 24 8 1596 9 10.1093/molbev/msm092 17488738

