# Supplementary figures and images for: Transcriptional regulation of the CRK/DUF26 group of Receptor-like protein kinases by ozone and plant hormones in Arabidopsis
Source: BMC Plant Biol. 2010 May 25;10:95. doi: 10.1186/1471-2229-10-95 (PMC3095361; doi:10.1186/1471-2229-10-95)

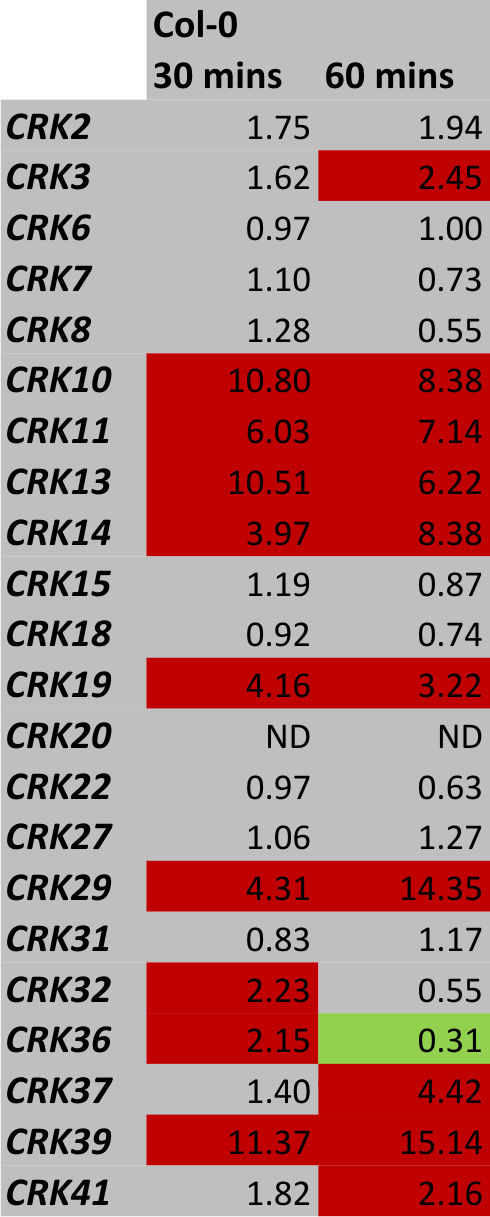

Supplement: Additional file 2 — Transcriptional regulation of the CRKs in response to flg22. 14-day old Arabidopsis Col-0 were treated with 100 nM flg22 and samples taken after 30 and 60 minutes (water-treated control samples have been harvested at the same time points in parallel). Expression of several CRKs was analyzed by qPCR. Transcript levels were calculated by comparison with the corresponding control plants. An expression level of one indicates no change in expression, increased expression is indicated by values larger than one while decreased expression is shown by values smaller than one. Increase in expression by 2-fold or higher is high-lighted in red and decrease in expression by 2-fold or more in green. [file 1471-2229-10-95-S2.PNG]
